# Early-Onset Creutzfeldt-Jakob Disease Mimicking Immune-Mediated Encephalitis

**DOI:** 10.3389/fneur.2018.00242

**Published:** 2018-04-10

**Authors:** Wietse A. Wiels, Stephanie Du Four, Laura Seynaeve, Anja Flamez, Thomas Tousseyn, Dietmar Thal, Miguel D’Haeseleer

**Affiliations:** ^1^Department of Neurology, Universitair Ziekenhuis Brussel, Centre for Neurosciences, Vrije Universiteit Brussel, Brussels, Belgium; ^2^Department of Pathology, Universitair Ziekenhuis Leuven, Leuven, Belgium; ^3^Translational Cell and Tissue Research Laboratory, Department of Imaging and Pathology, Universitair Ziekenhuis Leuven, Leuven, Belgium; ^4^Department of Neurosciences, Laboratory for Neuropathology, Katholieke Universiteit Leuven, Leuven, Belgium; ^5^Nationaal Multiple Sclerose Centrum, Melsbroek, Belgium

**Keywords:** Creutzfeldt-Jakob disease, VV1 type, progressive encephalomyelitis with rigidity and myoclonus, glycine receptor antibodies, dementia

## Abstract

**Objectives:**

The objective of this study is to explore the clinical, radiological, and pathological manifestations of a rare subtype of prion disease and their implication for differential diagnosis in case of an early onset neuropsychiatric deterioration.

**Methods:**

We discuss a patients’ clinical history, as well as the string of investigations and symptomatological evolution that finally led to a pathological diagnosis.

**Results:**

Our patient had the extremely rare VV1 type sporadic Creutzfeldt-Jakob disease (sCJD). We explain the differential diagnosis of progressive encephalomyelitis with rigidity and myoclonus and its implications for treatment.

**Conclusion:**

sCJD, especially the VV1 subtype, can present at an early age with an insidious psychiatric onset. Classical findings of prion disease—14-3-3 protein, PSWC on electroencephalography, and magnetic resonance imaging patterns—are not always present. The presence of neural autoantibodies does not always implicate pathogenicity in the presence of other neurological/neurodegenerative conditions.

## Clinical History

We present the case of a 33-year-old man who was referred to our neurology department from a mental hospital. Major difficulties emerged 6 months earlier, when the patient started to experience auditory hallucinations and personality changes. Disorganized, reclusive, and wandering behavior was noticed by his peers. Motor tasks became increasingly difficult, evolving into a situation in which the patient could no longer perform basic activities of daily living. In the preceding months, milder signs of depression and forgetfulness had been attributed to excessive use of marihuana and experimentation with other drugs, including khat and heroin.

An outpatient consultation already reported bradyphrenia, word-finding difficulties, ideatoric apraxia, and severe apathy—4 months before visiting our clinic. No signs of motor or gait dysfunction were noted at this moment. Routine blood analysis, brain magnetic resonance imaging (MRI), and electroencephalography (EEG) were normal. A schizophrenia spectrum disorder was suspected and the patient was admitted to residential psychiatric care. Poor response to treatment and further decline—into a state described as catatonia by mental health providers—warranted transfer to a neurological unit. Upon arrival in our hospital, the patient was cachectic, incontinent, and mute. Cranial nerve examination demonstrated vertical gaze palsy and saccadic slowing. There was severe axial rigidity and “Pisa”-type dystonia, with rigidity in the upper limbs and spasticity in the lower. Tendon reflexes were brisk and extensor plantar responses were bilaterally present. Further notable were choreiform and myoclonic jerks superimposed on prolonged spasms, present during rest but exacerbated by action. The patient exhibited extreme hyperekplexia and fell out of his chair when startled. Extensive heteroanamnesis did not lead to clues of unusual toxin exposure, apart from the illicit drugs mentioned above. No further particularities were present in his personal or family medical record.

## Technical Investigations

**Figure 1:**
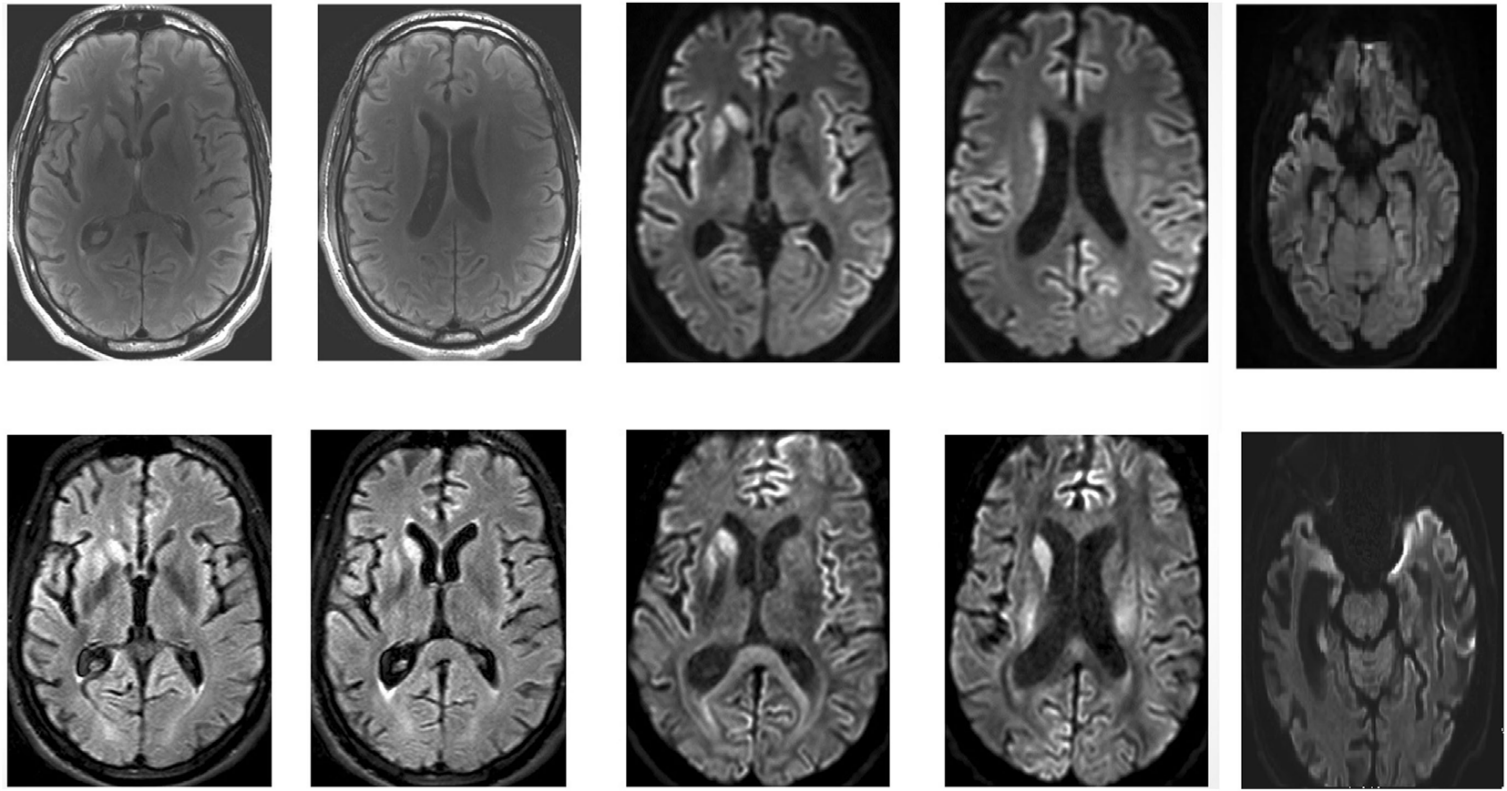
Magnetic resonance imaging of the patient’s brain. Top: April 2016. Bottom: May 2016. Asymmetric T2, fluid-attenuation inversion recovery (FLAIR), and diffusion-weighted (DWI) hyperintensities in the putamen and caudate, as well as frontal and temporal cortical regions of restricted diffusion are clearly visible.

Repeated brain MRI revealed asymmetric T2 hyperintensities in the putamen and caudate, as well as frontal and temporal cortical regions of restricted diffusion (Figure 1). Spinal cord imaging could not be interpreted due to movement artifacts. Full-body positron emission tomography was normal. EEG upon admission, with monthly controls thereafter, demonstrated generalized slowing, but did not show any periodic discharges. Routine blood analyses were normal, as were copper biochemistry and amino acid panels. Thyroid autoimmunity and antinuclear antibodies were excluded. Serologic screening for human immunodeficiency virus, treponemals, and borreliosis was negative. Cerebrospinal fluid analysis (CSF) demonstrated an elevated protein level (745 mg/dl) with normal cell count, immunofixation and 14-3-3 protein levels. Prion protein seeding activity could not be detected in CSF (through real-time-quaking induced conversion). The young age and prominent neuropsychiatric features of our patient further prompted investigation of variant CJD through tonsillar biopsy, which proved to be negative. A neurological autoantibody panel, using immunofixation on transfected cells and Western Blotting in serum and CSF, revealed no evidence of antibodies to NMDA-R, VGKC (LGI1/Caspr2), Hu, Ri, Yo, Ma1, Ma2, CV2/CRMP5, amphiphysin, Sox-1, Zic-4, DPPX, mGluR, GAD65, or Tr. It did, however, suggest the presence of low titers of glycine-receptor antibodies. Serum and CSF samples were initially tested at a dilution of 1/20. Fluorescence was then visually scored on a scale of 0–4. A score of 4 is the strongest staining, with 1.5 being considered as a low positive. Positive samples at 1/20 are further titrated to a 1/100 dilution and scored again. Our patient’s sample was positive at 1/20 with a visual score of 2. The samples were negative at 1/100 with a visual score of 0.5. Genetic screening for Huntington’s, dentatorubropallidoluysian atrophy, spinocerebellar ataxias, and Niemann-Pick disease C was negative. Prion protein gene (PRNP) analysis revealed a valine/valine (V/V) genotype for codon 129.

## Treatment and Follow-Up

Our differential diagnosis mainly consisted of prion disease and progressive encephalomyelitis with rigidity and myoclonus (PERM) with antiglycine receptor antibodies (1). Imaging and clinical course were not felt to be suggestive of a toxic encephalopathy connected to illicit drug use. We consecutively treated the patient with high-dose intravenous corticosteroids (1 g per day during 5 days, no effect), plasmapheresis (discontinuation after two sessions because of catheter sepsis), and monthly regimens of cyclophosphamide (four courses), but the patient’s neurological condition further deteriorated. A control brain MRI showed extensive and widespread cerebral atrophy. Immunosuppressive treatment was discontinued. Our patient became stuporous, had severe bulbar dysfunction and regressed into a fetal position. The patient died in a palliative care setting, some 16 months after the initial signs of disease.

## Diagnosis

**Figure 2:**
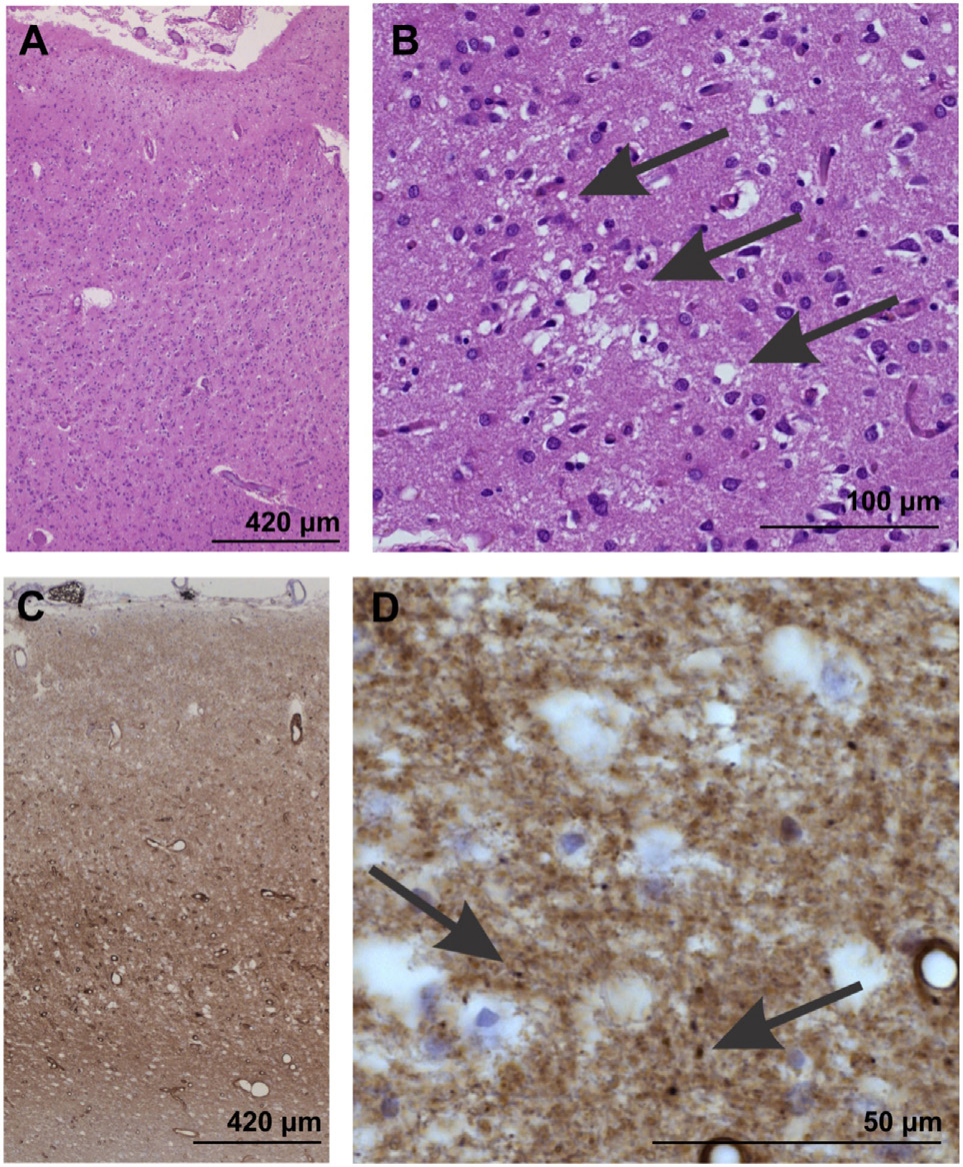
Neuropathological confirmation of Creutzfeldt-Jacob disease morphologically by neuron loss and astrogliosis in the temporal neocortex (area 35) as evident in the overview magnification of a hematoxylin and eosin-stained section **(A)**. Spongiform changes become visible at higher magnification [arrows in **(B)**]. **(C,D)** In the temporal neocortex of Brodmann area 35, the synaptic pattern of the pathological prion protein deposits become evident **(C)**. At higher magnification **(D)**, some more condensed, dot-like deposits can be seen (arrows), whereas no plaques or plaque-like lesion were seen. Prion protein antibody used: 3F4, proteinase K, and formic acid pretreatment, 1/100, Dako-Agilent, Santa-Clara, CA, USA.

**Figure 3:**
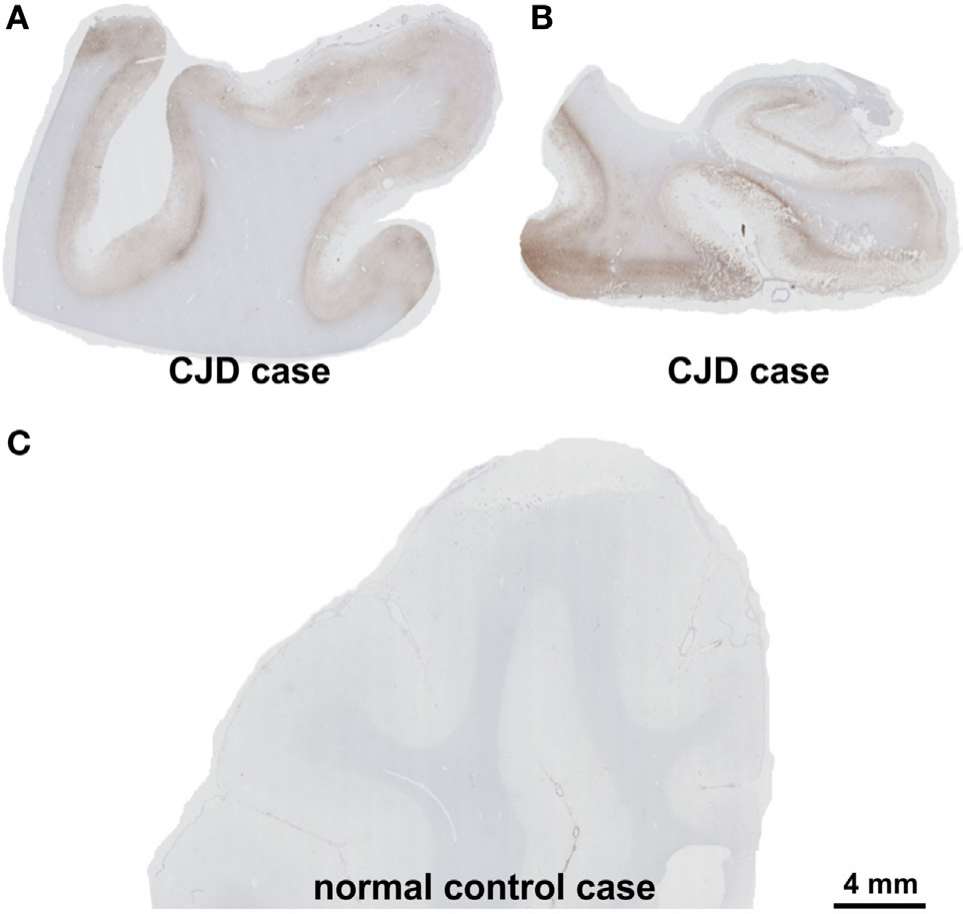
Presence of pathological prion protein proven by the detection of proteinase K-stable prion-protein aggregates in the frontal cortex **(A)** and the medial temporal lobe with the hippocampal formation **(B)** of the patient. In the control case, physiological prion protein was digested completely by proteinase K and the cortex showed no labeling **(C)**. Prion protein antibody used: 3F4, proteinase K, and formic acid pretreatment, 1/100, Dako-Agilent, Santa-Clara, CA, USA. Calibration bar in **(C)** valid for **(A,B)**. *Comment on the distribution pattern of pathological prion protein*. Pathological, proteinase K-stable prion protein was found in the neo- and allocortex and in the striatum whereas these deposits as well as the spongiform changes were less abundant in the cerebellum. This distribution pattern can be found in codon 129 VV1 type patients (2).

On autopsy, pathological proteinase K-stable prion protein aggregates were found in the neo- and allocortex, as well as in the striatum. These deposits as well as spongiform changes were less abundant infratentorially. This distribution pattern fits with that of sporadic Creutzfeldt-Jakob (sCJD) patients of the VV1 subtype (2) (Figures 2 and 3). Immunohistochemistry did not detect any IgG in brain parenchyma.

## Discussion

We present an unusual case of sCJD with insidious psychiatric onset in a young patient. The absence of classic CSF and EEG findings of prion disease posed additional diagnostic challenges. sCJD is classified according to PRNP genotype (combinations of methionine and/or valine) and pathogenic prion protein (PrPSc) molecular weight (21 and 19 kDa variants termed type 1 and type 2, respectively). The VV1 or so-called “early-onset” subtype is an extremely rare variant of sCJD (<1%), usually manifesting at a younger age and progressing at a slower pace (2–4). To our knowledge, this is the second reported case of VV1 sCJD with normal 14-3-3 protein levels in CSF; the other case coming from a series of 14 patients without additional clinical information (5). MRI abnormalities in the basal ganglia are considered unusual in this variant, whereas the absence of periodic sharp wave complexes on EEG, characteristic of classic sCJD, has been described in most published VV1 cases. A negative result on RT-QuIC can be considered very striking, as this test has a sensitivity and specificity that approaches 100% in the appropriate clinical context (6). Whether this finding reflects sheer coincidence or an emerging characteristic of the VV1 type sCJD, remains unclear. RT-QuIC is a relatively novel technique and, to the best of our knowledge, no previous test results in confirmed cases of this rare subtype have been described so far.

The presence of autoantibodies in our patient initially suggested another diagnosis—PERM. The expanding spectrum of immune-mediated encephalopathies has been a major addition to the field of neurology in the past decade. Because of the potentially treatable nature of these disorders, the threshold for consideration is often low in clinical practice. While detectable neural autoantibodies in healthy controls are very rare, positive assays have occasionally been described in other neurological diseases, including CJD, possibly reflecting neural epitope exposure. Nevertheless, their presence in sCJD is highly unusual, often with equivocal test results (7, 8). One single case of (unspecified) sCJD with glycine-receptor autoantibodies has been previously described, in a 68-year-old male who also had antibodies against the VGKC-complex (9, 10). In our case, we did not find IgG accumulation in the brain—suggesting that these antibodies did not impact disease course. While some toxic encephalopathies can mimic the clinical presentation of prion disease, and heroin abuse has been linked to premature neurodegeneration (11), a direct link between illicit drug use and prion disease has yet to be suggested.

Most reported cases of diagnostic confusion between sCJD and immune-mediated encephalitis with antineuronal antibodies had MRI findings that were either normal or considered typical for limbic encephalitis (12). Overall, brain MRI findings in our subject were considered as being suggestive for sCJD. In addition to the basal ganglia abnormalities, widespread diffusion restriction of the frontotemporal cortex was noticeable. Early and prominent neuropsychiatric features in other sCJD cases have been attributed to lesional involvement of limbic structures (13). Antibody titers correlate with disease severity in PERM but were low in our subject. Furthermore, antibodies may not always be disease-specific and many PERM cases appear to be seronegative (1). Congruence with the patient’s clinical picture and concern for therapeutic possibilities, however, prompted our consideration of this diagnostic option. It was *a priori* decided that the patient’s clinical evolution (rather than follow-up antibody titers) would best reflect response to immunological treatments.

Our case highlights the heterogeneity of sCJD and supports diagnostic awareness in cases of rapidly progressive dementia with suggestive imaging, even in young adults and/or when other typical features are lacking. Biochemical overlap with immune-mediated disorders may cause further diagnostic confusion for clinicians and must be interpreted with care.

## Ethics Statement

This study was carried out in with written informed consent from the subject’s family members. They gave their written informed consent for publication in accordance with the Declaration of Helsinki and the late patients’ will.

## Author Contributions

WW: manuscript, patient care, submission, and concept. SF: patient care, concept, critical review of manuscript, and supplementary material. LS: manuscript, patient care, and critical review of manuscript. AF: patient care and critical review of manuscript. TT: critical review of manuscript, supplementary material, and autopsy. DT: critical review of manuscript, supplementary neuropathological data, and autopsy. MD: case supervision, critical review of manuscript, and patient care.

## Conflict of Interest Statement

The authors declare that the research was conducted in the absence of any commercial or financial relationships that could be construed as a potential conflict of interest.
